# Nuclear Receptor 4A1 (NR4A1) as a Drug Target for Renal Cell Adenocarcinoma

**DOI:** 10.1371/journal.pone.0128308

**Published:** 2015-06-02

**Authors:** Erik Hedrick, Syng-Ook Lee, Gyungeun Kim, Maen Abdelrahim, Un-Ho Jin, Stephen Safe, Ala Abudayyeh

**Affiliations:** 1 Department of Veterinary Physiology and Pharmacology, Texas A&M University, College Station, TX, United States of America; 2 Department of Food Science and Technology, Keimyung University, Daegu, Republic of Korea; 3 Institute of Biosciences and Technology, Texas A&M Health Sciences Center, Houston, TX, United States of America; 4 Department of Internal Medicine, Baylor College of Medicine, Houston, TX, United States of America; 5 Department of General Internal Medicine, University of Texas MD Anderson Cancer Center, Houston, TX, United States of America; Lunenfeld-Tanenbaum Research Institute, CANADA

## Abstract

The orphan nuclear receptor NR4A1 exhibits pro-oncogenic activity in cancer cell lines. NR4A1 activates mTOR signaling, regulates genes such as thioredoxin domain containing 5 and isocitrate dehydrogenase 1 that maintain low oxidative stress, and coactivates specificity protein 1 (Sp1)-regulated pro-survival and growth promoting genes. Transfection of renal cell carcinoma (RCC) ACHN and 786-O cells with oligonucleotides that target NR4A1 results in a 40–60% decrease in cell proliferation and induction of apoptosis. Moreover, knockdown of NR4A1 in RCC cells decreased bcl-2, survivin and epidermal growth factor receptor expression, inhibited of mTOR signaling, induced oxidative and endoplasmic reticulum stress, and decreased *TXNDC5* and *IDH1*. We have recently demonstrated that selected 1,1-bis(3'-indolyl)-1-(*p*-substituted phenyl)methane (C-DIM) compounds including the *p*-hydroxyphenyl (DIM-C-pPhOH) and *p*-carboxymethyl (DIM-C-pPhCO_2_Me) analogs bind NR4A1 and act as antagonists. Both DIM-C-pPhOH and DIM-C-pPhCO_2_Me inhibited growth and induced apoptosis in ACHN and 786-O cells, and the functional and genomic effects of the NR4A1 antagonists were comparable to those observed after NR4A1 knockdown. These results indicate that NR4A1 antagonists target multiple growth promoting and pro-survival pathways in RCC cells and in tumors (xenograft) and represent a novel chemotherapy for treating RCC.

## Introduction

Kidney cancer is a complex and heterogenous disease and clear cell renal cell adenocarcinoma (RCC) is the most common sub-type and represents approximately 75% of renal parenchymal tumors [1–3]. The incidence of RCC has been increasing and it is estimated that in 2013 over 65,000 new cases will be diagnosed and 13,680 kidney cancer patients will die from this disease [4]. Early stage RCC patients with localized tumors have a good prognosis after surgical removable of the primary tumor; however, approximately 30% of all patients first diagnosed with RCC already have metastatic disease [1]. Patients with RCC are unusually resistant to radio and cytotoxic drug therapies compared to responses observed for other solid tumors, and immunotherapies have provided some limited benefits for patients with RCC metastasis [5–8]. Recent developments of targeted therapies for treating RCC have primarily focused on clinical applications of receptor tyrosine kinase inhibitors, neutralizing antibodies against vascular endothelial growth factor (VEGF) and mTOR pathway inhibitors [9–13].

**Fig 1:**
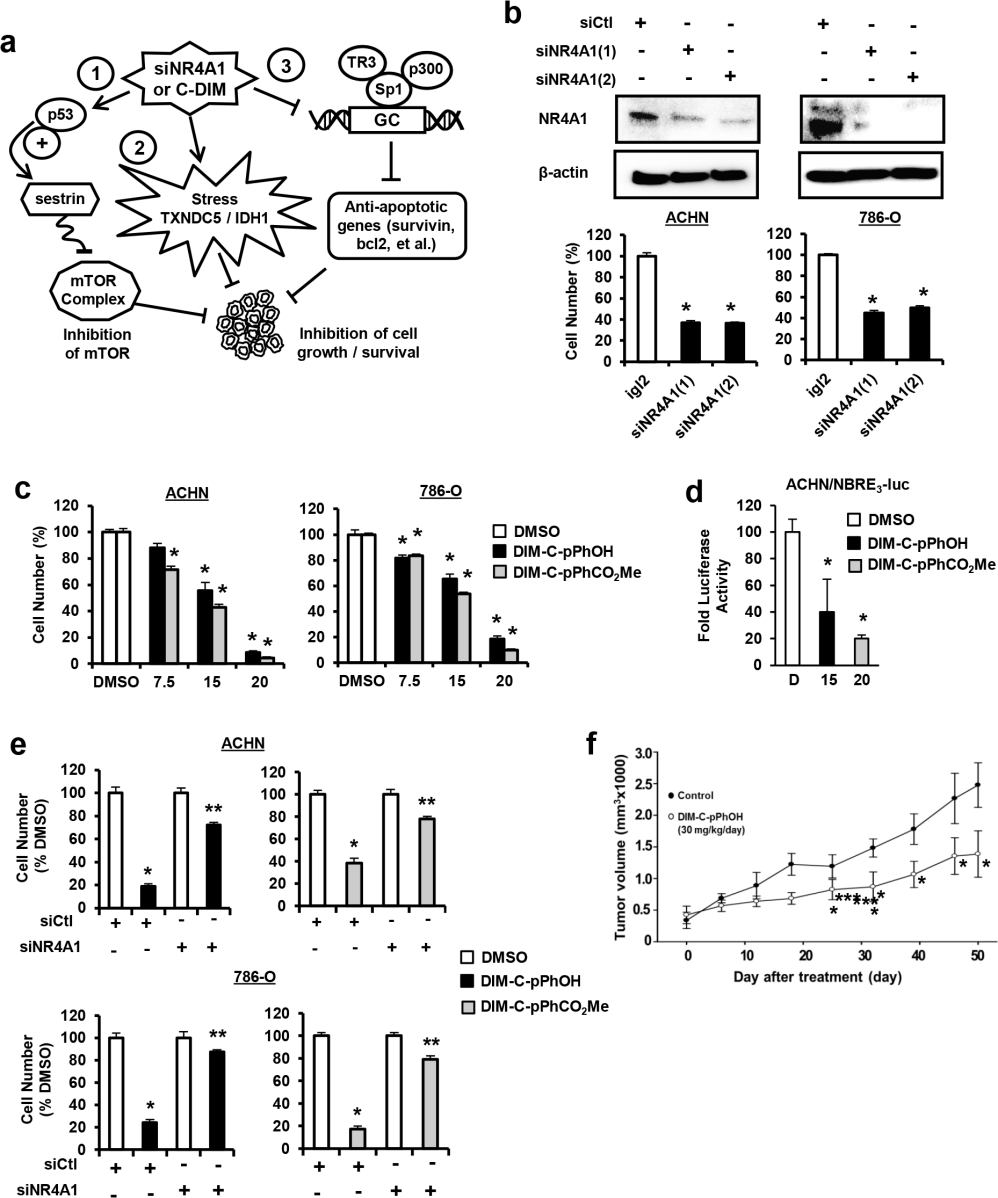
NR4A1 plays a role in RCC proliferation. (A) Pathways activated by NR4A1 and targeted by NR4A1 antagonists. (B) Cells were transfected with two different oligonucleotides targeting NR4A1 [NR4A1(1) and NR4A1(2)] and after 72 hr, the number of cells were determined as outlined in the Materials and Methods. (C) Cells were treated with DIM-C-pPhOH and DIM-C-pPhCO_2_Me and cell numbers were determined after treatment for 24 hr. (D) Cells were transfected with NBRE_3_-luc and 40 ng FLAG-NR4A1, treated with DMSO, DIM-C-pPhOH (20 μM) and DIM-C-pPhCO_2_Me (15 μM), and luciferase activity was determined as described [17]. (E) Cells were transfected with siCtl (non-specific) or siNR4A1 and treated with DIM-C-pPhOH and DIM-C-pPhCO_2_Me and cell numbers were determined. siNR4A1 alone decreased cell proliferation as indicated in (B) and this value was set at 100% to determine the effects of C-DIMs in cells after loss of NR4A1. (F) Athymic nude mice bearing ACHN cells as xenografts were treated with 30 mg/kg/d DIM-C-pPhOH and tumor volumes were determined. Results (C—E) are means ± SE for at least 3 separate determinations and significantly (p < 0.05) decreased growth/volume is indicated (*). Significant (p < 0.05) attenuation of C-DIM-induced growth or luciferase activity or after transfection with siNR4A1 is also indicated (**).

Studies in this laboratory have identified 1,1-bis(3'-indolyl)-1-(*p*-substituted phenyl)methanes (C-DIMs) as novel antineoplastic agents that bind and act as antagonists of the orphan nuclear receptor 4A1 (NR4A1, TR3, Nur77) which exhibits pro-oncogenic activity in several solid tumors [14–17]. NR4A1 knockdown or treatment with the *p*-hydroxyphenyl-derived (DIM-C-pPhOH) NR4A1 antagonist in pancreatic, lung and colon cancer cell lines show that NR4A1 regulates at least three pathways that are important for cancer cell proliferation and survival (Fig 1A). NR4A1 regulates expression of pro-survival (survivin, *bcl-2*) and growth promoting (*EGFR*) genes through interaction with Sp1 bound to their corresponding proximal GC-rich promoters [14]. NR4A1 binds to and inactivates p53 and p53-regulated sestrin 2 resulting in activation of mTOR. NR4A1 and wild-type p53. NR4A1 also regulates expression of genes such as thioredoxin domain containing 5 (*TXNDC5*) and isocitrate dehydrogenase 1 (*IDH1*) to maintain low oxidative stress in cancer cells [16]. Thus, inactivation of NR4A1 by the receptor antagonist DIM-C-pPhOH or the corresponding *p*-carboxymethyl analog (DIM-C-pPhCO_2_Me) decreases expression of genes associated with cell proliferation and survival, induces oxidative stress, and inhibits mTOR in cancer cell lines [17]. In this study, we show that NR4A1 is also expressed and exhibits pro-oncogenic activity in ACHN and 786-O kidney cancer cell lines and treatment with DIM-C-pPhOH and related NR4A1 antagonists inhibit cell growth and mTOR signaling, induce apoptosis and cellular stress (Fig 1A), suggesting that NR4A1 is a potential novel drug target for treating RCC patients that overexpress this receptor.

## Methods and Materials

### Cell lines, antibodies, proliferation and immunofluorescence

ACHN and 786-O human kidney cancer cell lines were purchased from American Type Culture Collection (Manassas, VA). Cells were maintained 37°C in the presence of 5% CO_2_ in Dulbecco’s modified Eagle’s medium/Ham’s F-12 or RPMI-1460 medium (Sigma-Aldrich, St. Louis, MO) with 10% fetal bovine serum. Sources of the antibodies used are summarized in Table 1. Cells were visualized under an EVOS fl, Fluorescence microscope, from Advanced Microscopy Group using a multiband filter set for FITC, rhodamine, and DAPI. The C-DIM compounds were prepared as previously described [9–11]. ACHN and 786-O kidney cancer cells (1.0 x 10^5^ per well) were plated in 12-well plates and allowed to attach for 24 hr, and treatment or knockdown experiments and immunofluorescence of NR4A1 was determined essentially as described [15–17].

**Table 1:** Antibodies and sources.

Antibodies	Sources
Sp1	Millipore (Temecula, CA)
Sestrin 2 (SESN2), bcl2, CHOP, ATF4, IDH1, EGFR	Santa Cruz Biotech (Santa Cruz, CA)
Nur77 (D63C5)XP, caspase 3, cleaved poly (ADP)ribose polymerase (c-PARP), phospho-mTOR, mTOR, phospho-AMPKα, AMPKα, phospho-p70S6K, p70S6K, phospho-S6RP, S6RP, phospho-4EBP1, 4EBP1, and survivin	Cell Signaling Technologies (Danvers, MA)
TXNDC5	Genetex (Irvine, CA)
Phospho-PERK	Biolegend (San Diego, CA)

### Annexin V staining and ROS induction

ACHN and 786-O kidney cancer cells (1.0 x 10^5^ per well) were seeded and allowed to attach for 24 hr. The medium was then changed to DMEM/Ham F-12 medium contained 2.5% charcoal-stripped fetal bovine serum, and cells were treated and analyzed for Annexin V staining essentially as described [14]. Cellular ROS levels were ascertained using the cell permeable probe CM-H2DCFDA (5-(and-6)-chloromethyl-2'7'-dichlorodihydrofluorescein diacetate acetyl ester) from Invitrogen (Grand Island, NY).

### Western blot analysis

Cells were seeded and subsequently treated with varying concentrations of DIM-C-pPhOH or DIM-C-pPhCO_2_Me for 24 hr or transfected with 100 nM siNR4A1 for 72 hr, and whole cell lysates were analyzed by western blot analysis essentially as described using β-actin as the loading control [14–17].

### Small interfering RNA interference and transactivation assays

Knockdown of NR4A1 in ACHN and 786-O cells was carried out using Lipofectamine 2000 reagent according to the manufacture's protocol. Small inhibitory RNAs and GL2 (non-specific oligonucleotide) were prepared or purchased from Sigma-Aldrich (St. Louis MO), and siRNA complexes used in the study are as follows: siGL2-5', CGU ACG CGG AAU ACU UCG A; siNR4A1 (1)-SASI_Hs02_00333289; siNR4A1 (2)-SASI_Hs01_00182072. ACHN RCC cells were plated on 12-well plates at 5 x 10^4^ cells per well in DMEM supplemented with 2.5% charcoal-stripped FBS. After 24 hr, various amounts of DNA [NBRE_3_-luc (400 ng), FLAG-NR4A1 (40 ng)] were cotransfected into each well by Lipofectamine 2000 reagent (Invitrogen, Carlsbad, CA) according to the manufacturer's protocol. After 6 hr of transfection, cells were treated with 2.5% stripped DMEM containing either DMSO, DIM-C-pPhOH (20 μM) or DIM-C-pPhCO_2_Me (15 μM) for 18 hr. Cells were then analyzed for luciferase activity as previously described [17].

### Ethics Statement

Animal Care and Maintenance: The Animal Use Protocol (#IACUC 2013–0251) was approved by the Texas A&M University Institutional Animal Care and Use Committee (IACUC). Mice were housed in the Laboratory Animal Resources and Research Facility approved by the Texas A&M University IACUC at Texas A&M University, College Station, TX, and were attended by an animal care group and veterinarian. Comfort: Necessary steps were taken to minimize the pain and discomfort of all animals utilized in these studies. A scruff hold or gentle manual restraint was utilized to restrain mice for all procedures. Euthanasia: The mice were euthanized by asphyxiation (inhalation of CO_2_ in a small chamber). This method is consistent with recommendations of the Panel of Euthanasia of the American Veterinary Medical Association.

### Orthotopic xenograft model

Male athymic nude mice (Foxn1nu, aged 6–7 weeks) were purchased from Harlan (Indianapolis, IN) and housed under specific pathogen-free conditions. ACHN cells (10^7^ cells/150 μl) in Matrigel were injected into the flanks of individual mice (6 per group) and after 7 days when tumors were palpable, mice were randomized into two groups and dosed by oral gavage with either corn oil or DIM-C-pPhOH (30 mg/kg/day) for 50 days. The mice were weighed, and tumor size was measured twice a week with calipers to permit calculation of tumor volumes, V×L×W^2^/2, where L and W were length and width. Tumor lysates were obtained and analyzed for protein expression by western blots.

### Statistical analysis

Statistical significance of differences between the treatment groups was determined by student’s *t* test. The results are expressed as means with error bars representing 95% confidence intervals for 3 experiments for each group unless otherwise indicated, and a *P* value less than 0.05 was considered statistically significant. All statistical tests were 2-sided.

## Results

### NR4A1 antagonists inhibit RCC cell proliferation and induce apoptosis

**Fig 2:**
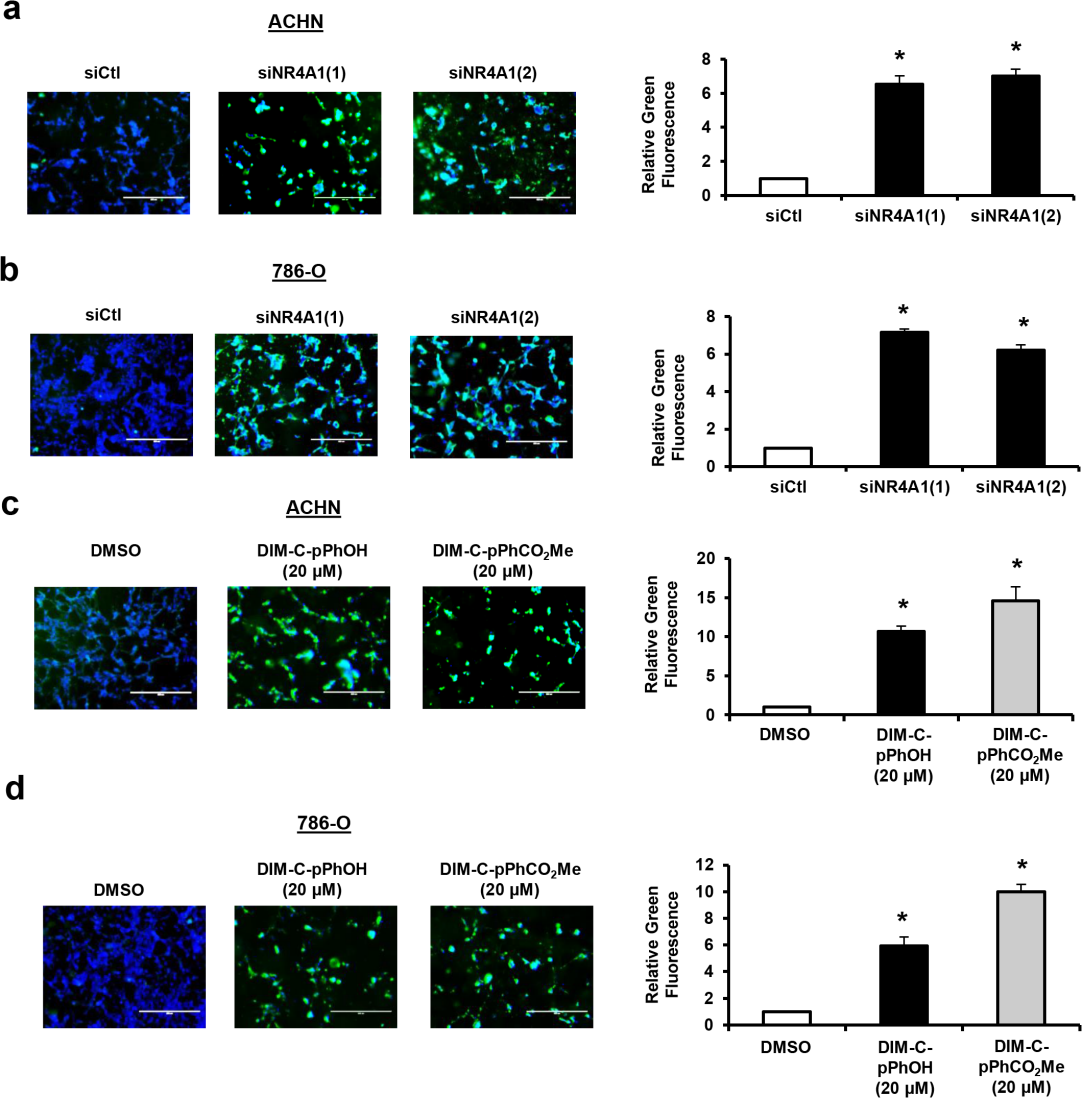
NR4A1 knockdown and C-DIM/NR4A1 antagonists induce apoptosis in RCC cells. ACHN (A) or 786-O (B) cells were transfected with siNR4A1(1) and siNR4A1(2) and Annexin V staining was determined as outlined in the Materials and Methods. ACHN (C) and 786-O (D) cells were treated with 20 μM DIM-C-pPhOH or DIM-C-pPhCO_2_Me for 24 hr and Annexin V staining was determined. Results are means ± SE for 3 replicated determinations and significant (p<0.05) induction of Annexin V staining is indicated (*).

Fig 1A summarizes the growth-promoting and survival pathways that can be targeted by NR4A1 antagonists in lung, pancreatic and colon cancer cells [14–17], and this study investigates these pathways in RCC cells and the role of C-DIM/NR4A1 antagonists as inhibitors of these pathways. ACHN and 786-O RCC cell lines are p53-positive and mutant cell lines, respectively, and in cells transfected with two different oligonucleotides that target NR4A1 (siNR4A1), there was a significant 50–60% decrease in proliferation of both cell lines (Fig 1B). Moreover, treatment of these cells with 0–20 μM of the NR4A1 antagonists DIM-C-pPhOH or DIM-C-pPhCO_2_Me also significantly decreased cell proliferation (Fig 1C). IC_50_ values for both compounds in ACHN cells were 13.6 and 11.7 μM, respectively, and in 786-O cells the values were 13.0 and 13.4 μM, respectively. ACHN cells were transfected with an NBRE-luc construct containing 3 monomer binding sites and both DIM-C-pPhOH and DIM-C-pPhCO_2_Me significantly decreased luciferase activity (Fig 1D) as previously described in colon cancer cells [17], demonstrating NR4A1 antagonist activity in this transactivation assay. The growth inhibitory effects of DIM-C-pPhOH and DIM-C-pPhCO_2_Me in ACHN and 786-O cells were significantly decreased after knockdown of NR4A1 by RNAi, thus demonstrating a role for NR4A1 in mediating the growth inhibitory effects of C-DIM/NR4A1 antagonists (Fig 1E). Moreover, treatment of athymic nude mice bearing ACHN cells as xenografts with DIM-C-pPhOH (30 mg/kg/d) for 50 days also resulted in a significant inhibition of tumor growth (Fig 1F) and complemented results of the *in vitro* studies. Thus, both knockdown of NR4A1 by RNAi or treatment with C-DIM/NR4A1 antagonists inhibited RCC cell and tumor growth. Transfection of ACHN and 786-O cells with two different siNR4A1 oligonucleotides also increased Annexin V staining (Fig 2A and 2B) which is a marker of apoptosis. We also observed that both DIM-C-pPhOH and DIM-C-pPHCO_2_Me induced Annexin V staining in ACHN and 786-O cells (Fig 2C and 2D, respectively), confirming that C-DIM/NR4A1 antagonists induce apoptosis in RCC cell lines. Moreover, in S1 Fig, we also show that siNR4A1 and C-DIM/NR4A1 antagonists also induce cleavage of caspases 7 and 8 in ACHN and 786-O cells.

**Fig 3:**
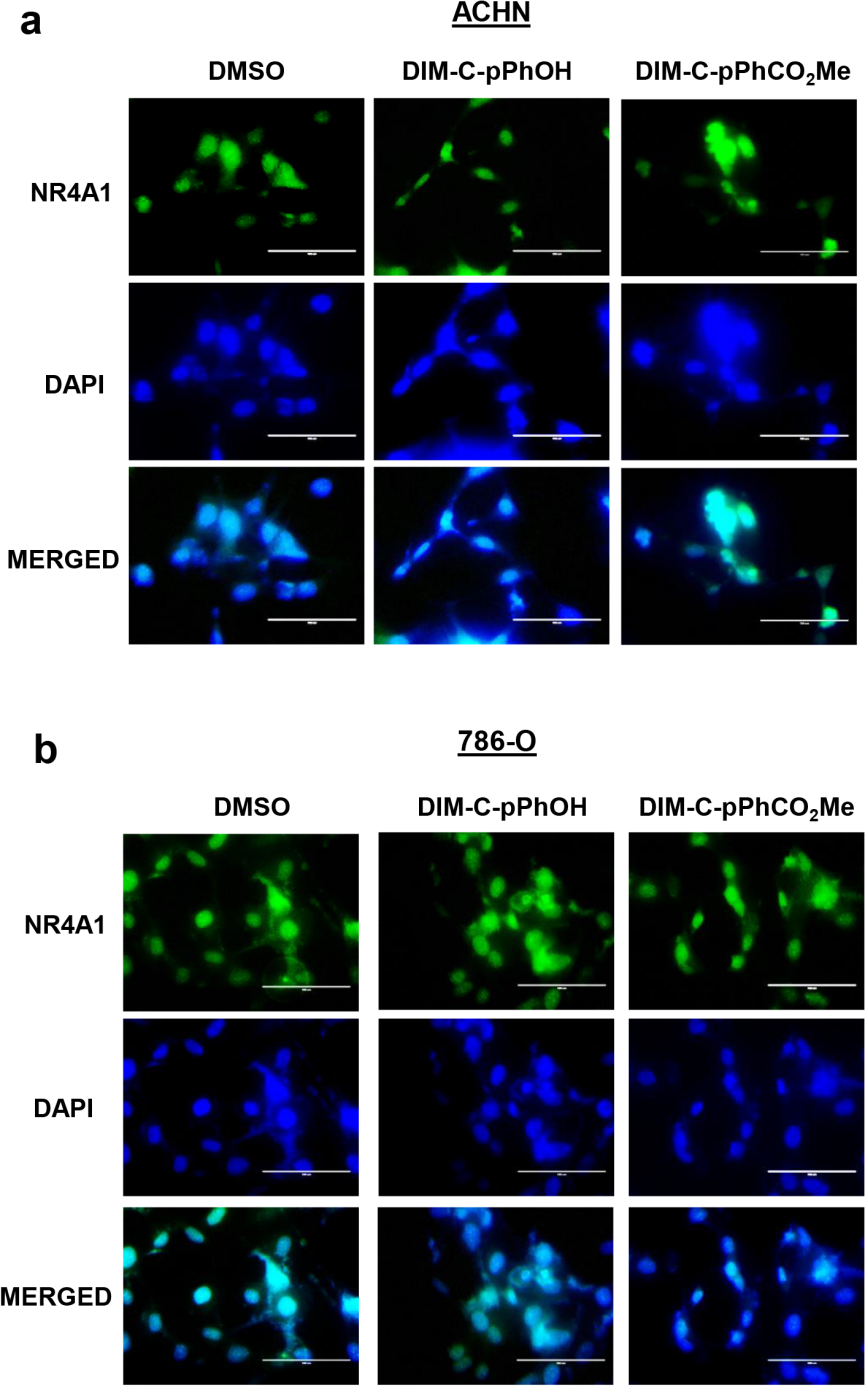
C-DIM/NR4A1 antagonists target nuclear NR4A1. ACHN (A) and 786-O (B) cells were treated with 20 μM DIM-C-pPhOH and DIM-C-pPhCO_2_Me. Cells were immunostained with NR4A1 antibodies or DAPI and images merged as outlined in the Materials and Methods.

Previous studies show that many apoptosis inducers that act through NR4A1 induce nuclear export of the receptor which subsequently forms a pro-apoptotic complex with the mitochondrial bcl-2 protein [18–20]. In contrast, our studies show that C-DIMs act through nuclear NR4A1 in cancer cells [14–17]. ACHN and 786-O cells were treated with DIM-C-pPhOH and DIM-C-pPHCO_2_Me and after 24 hr, cells were stained with NR4A1 antibodies and DAP1 and the results show that DAP1 and the NR4A1 immunostaining were co-localized in the nucleus, demonstrating that the C-DIM/NR4A1 antagonists act through the nuclear receptor (Fig 3).

### Sp-regulated survival genes

**Fig 4:**
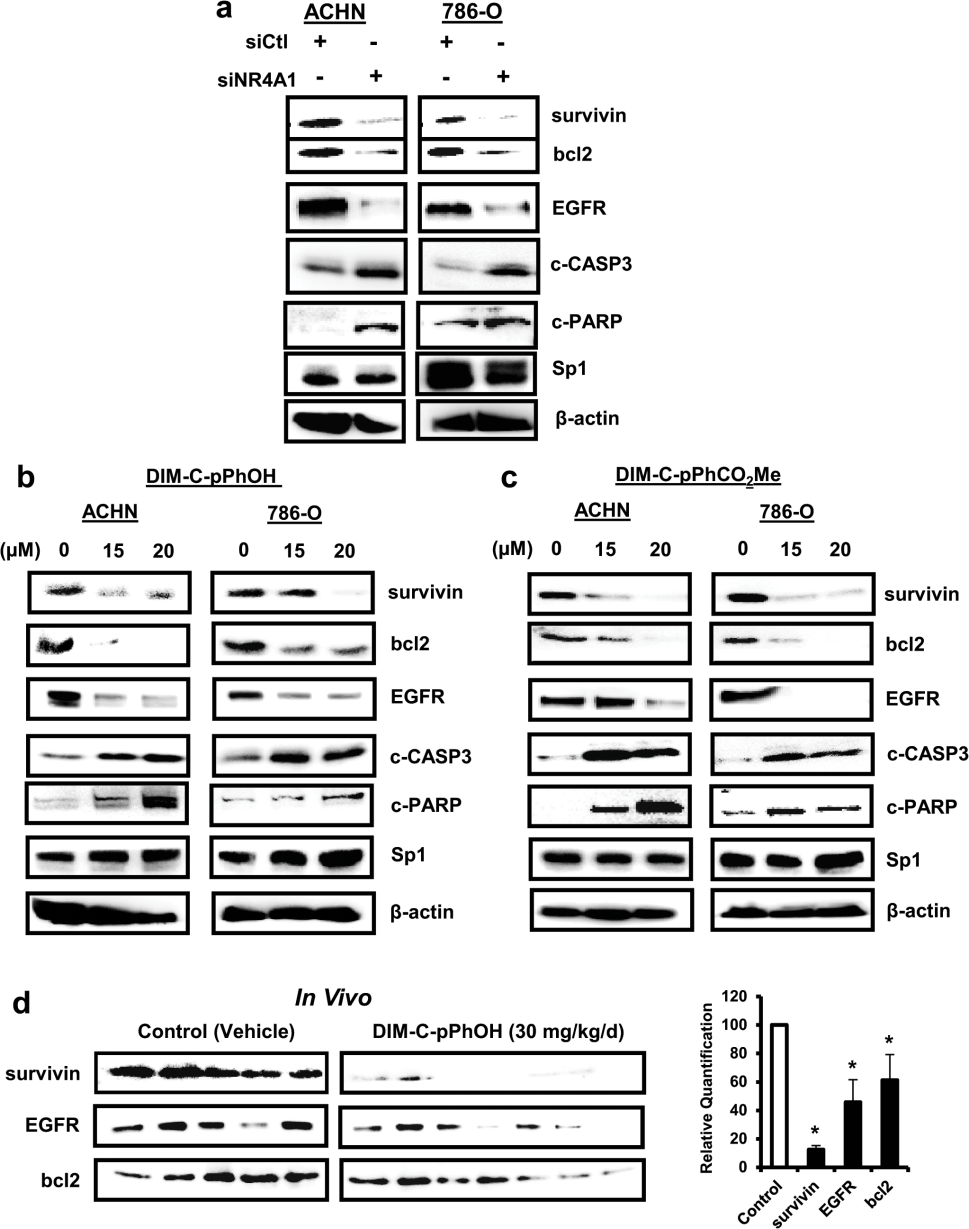
NR4A1 plays a role in expression of Sp-regulated growth promoting and survival genes. (A) Cells were transfected with siNR4A1 and whole cell lysates were analyzed by western blots as outlined in the Materials and Methods. Cells were treated with DIM-C-pPhOH (B) or DIM-C-pPhCO_2_Me (C) and after 24 hr, whole cell lysates were analyzed by western blots. (D) Western blot analysis of tumor lysates from athymic nude mice bearing ACHN xenografts and treated with vehicle (control) or DIM-C-pPhOH (30 mg/kg/d) was also determined. Band intensities were quantitated relative to β-actin (means ± SE) and significantly decreased staining intensities are indicated (*; p<0.05).

Previous studies showed that NR4A1 in combination with p300 activated Sp-regulated genes such as survivin, bcl-2 and EGFR in cancer cells [14–17]. Transfection of ACHN and 786-O cells with siNR4A1 decreased expression of survivin, bcl-2 and EGFR and this was accompanied by increased PARP cleavage (primarily in ACHN cells), a marker of apoptosis (Fig 4). Similar results were observed in both RCC cell lines after treatment with DIM-C-pPhOH (Fig 4B) or DIM-C-pPhCO_2_Me (Fig 4C), confirming that the NR4A1 antagonists inhibited NR4A1-regulated expression of survivin, bcl-2 and EGFR in ACHN and 786-O cells as previously reported in pancreatic, lung and colon cancers [14–17]. Thus, cells transfected with siNR4A1 or treated with C-DIM/NR4A1 antagonists induced multiple markers of apoptosis including increased Annexin V staining, cleavage of caspases 3, 7, 8 and PARP and this was accompanied by downregulation of the anti-apoptotic genes survivin and bcl-2. In addition, we also observed decreased expression of survivin, bcl-2, EGFR and induced PARP cleavage in tumor lysates from nude mice bearing ACHN cells as a xenograft and treated with DIM-C-pPhOH (30 mg/kg/d) (Fig 4D).

### siNR4A1 and C-DIM/NR4A1 antagonist induce stress in RCC cells

**Fig 5:**
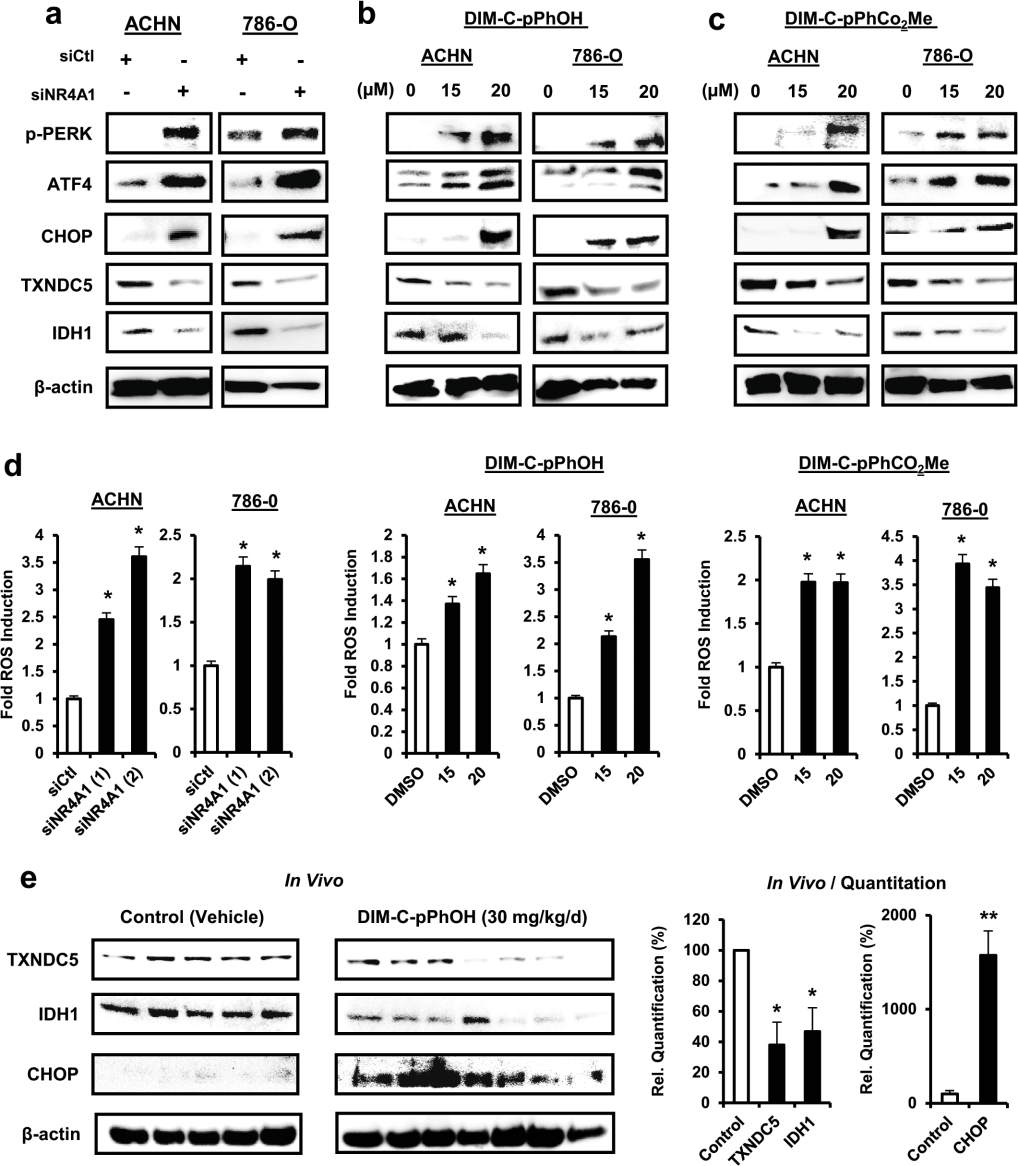
siNR4A1 and C-DIM/NR4A1 antagonists induce stress in RCC cells. Cells were transfected with siNR4A1 (A) or treated with DIM-C-pPhOH (B) or DIM-C-pPhCO_2_Me (C) and whole cell lysates were analyzed by western blots. (D) After treatment of cells as described in (A)—(C), ROS was measured using the cell permeant CM-H2DCFDA probe as outlined in the Materials and Methods. Significant induction (p<0.05) of ROS is indicated (*). (E) Tumor lysates from athymic nude mice bearing ACHN cells as xenografts and treated with vehicle control or DIM-C-pPhCO_2_Me were analyzed by western blots and individual bands were quantitated (relative to β-actin). Significant (p<0.05) induction of ROS is indicated (*).

It has been shown that NR4A1 maintains low levels of stress in cancer cells by regulating expression of TXNDC5 and IDH1 (Fig 1A) that in turn maintain high levels of cellular reducing agents [16, 17]. Transfection of ACHN and 786-O cells with siNR4A1 decreased expression of TXNDC5 and IDH1 in both cell lines and this was accompanied by increased expression of CHOP, ATF4 and phospho-PERK which are markers of ER stress (Fig 5A). Treatment of ACHN and 786-O cells with DIM-C-pPhOH (Fig 5B) or DIM-C-pPhCO_2_Me (Fig 5C) also decreased expression of TXNDC5 and IDH1 and induced markers of ER stress (CHOP, ATF4 and p-PERK). Moreover, siNR4A or treatment with the C-DIM/NR4A1 antagonists also induced ROS as determined using the cell permeant probe CM-H2DCFCA (Fig 5D). In addition, DIM-C-pPhOH (30 mg/kg/d) also decreased expression of TXNDC5 and IDH-1 and induced CHOP expression in tumors from athymic nude mouse xenografts (Fig 5E), demonstrating that C-DIM/NR4A1 antagonists-dependent inhibition of RCC cell and tumor growth is due, in part, to induction of stress (Fig 1A).

### siNR4A1 and C-DIM/NR4A1 antagonists inhibit mTOR

**Fig 6:**
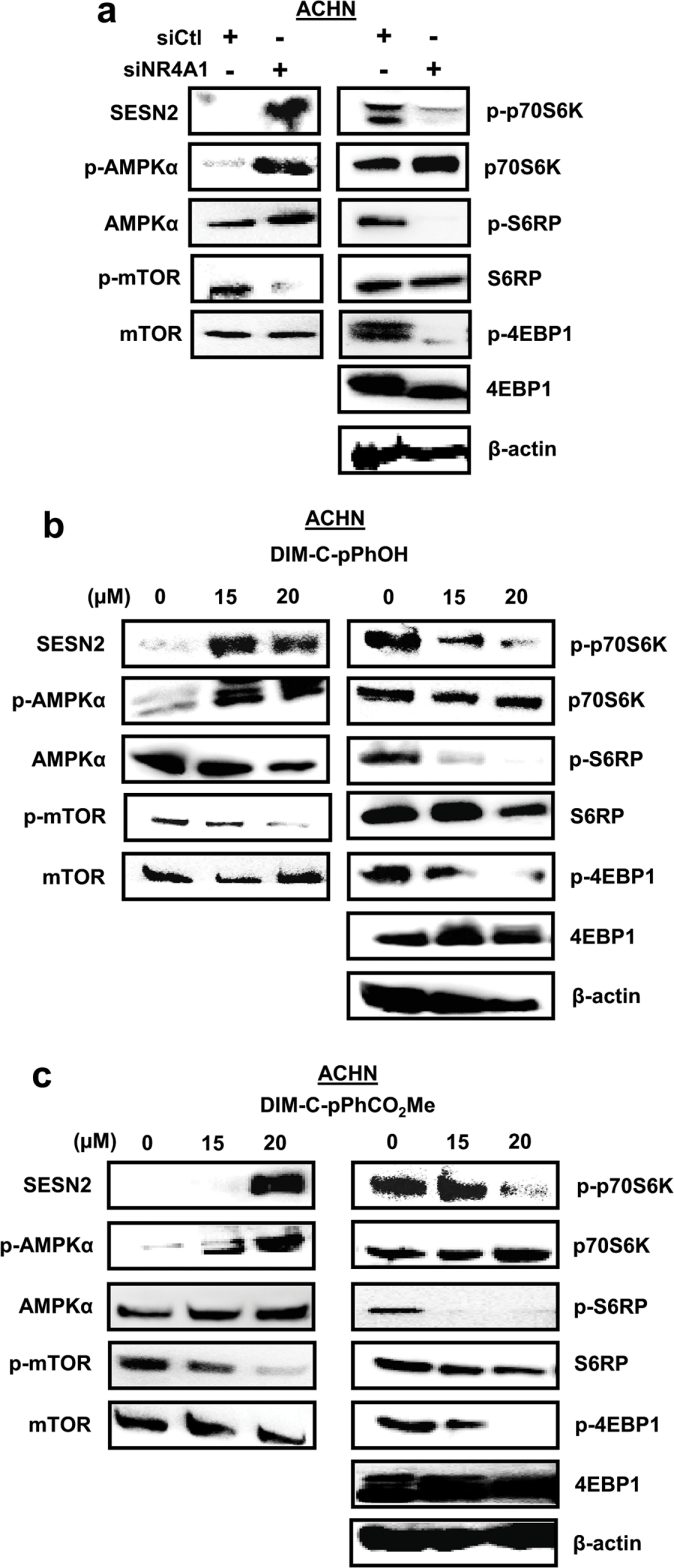
siNR4A1 and NR4A1 antagonist inhibit mTOR in ACHN cells. Cells were transfected with siNR4A1 (A) and treated with DIMI-C-pPhOH (B) and DIM-C-pPhCO_2_Me (C), and whole cell lysates were analyzed by western blots as outlined in the Materials and Methods.

**Fig 7:**
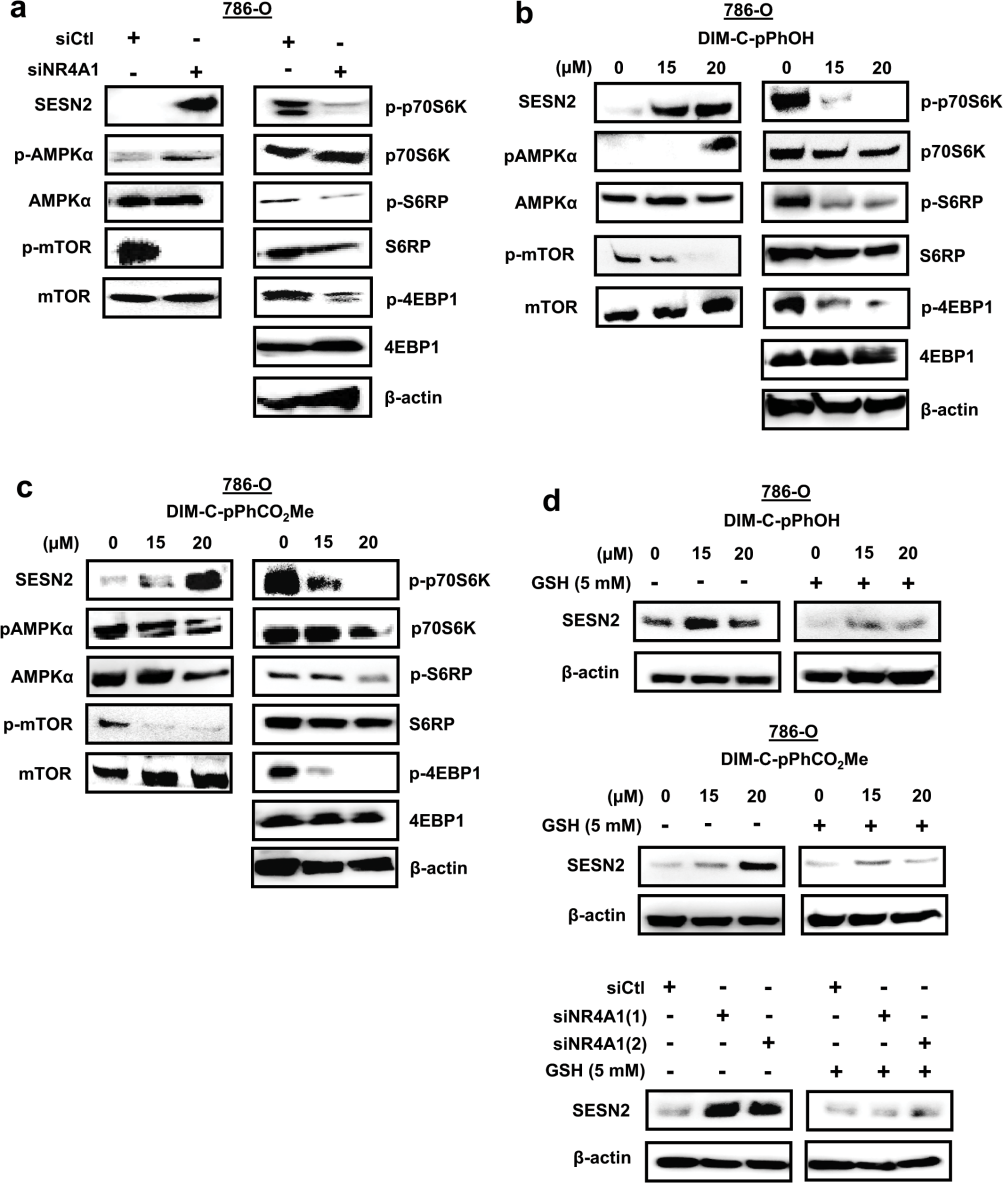
siNR4A1 and NR4A1 antagonist inhibit mTOR in 786-O cells. Cells were transfected with siNR4A1 (A) and treated with DIMI-C-pPhOH (B) and DIM-C-pPhCO_2_Me (C), and whole cell lysates were analyzed by western blots as outlined in the Materials and Methods. (D) Induction of sestrin 2 by western blots was determined in 786-O cells treated with DIM-C-pPhOH and DIM-C-pPhCO_2_Me or transfected with siNR4A1 in the presence or absence of 5 mM GSH.

NR4A1 binds and inactivates p53 [21], and treatment with siNR4A1 or C-DIM/NR4A1 antagonists results in p53-dependent induction of sestrin 2 which activates AMPKα and inhibits mTOR [15, 17]. Knockdown of NR4A1 by RNAi in ACHN cells that express wild-type p53 resulted in the induction of sestrin 2, activation of AMPKα, and inhibition of phospho-mTOR; this was also accompanied by decreased activation/phosphorylation of mTOR-regulated p70S6K, S6RP and 4EBP1 (Fig 6A). Treatment of ACHN cells with the NR4A1 antagonists DIM-C-pPhOH (Fig 6B) and DIM-C-pPhCO_2_Me (Fig 6C) also induced sestrin 2, activated AMPKα and inhibited activation of mTOR and downstream kinases and these results are consistent with previous studies in other cancer cells expressing wild-type p53 [15, 17]. The 786-O cell line expresses a mutant p53; however, transfection of these cells with siNR4A1 also resulted in the induction of sestrin 2 and inactivation of mTOR and mTOR signaling (Fig 7A). Moreover, similar results were observed after treatment of 786-O cells with the NR4A1 antagonists DIM-C-pPhOH (Fig 7B) and DIM-C-pPhCO_2_Me (Fig 7C). It has previously been reported that sestrin 2 is induced by other factors including oxidative stress [22], and Fig 7D shows that induction of sestrin 2 by siNR4A1, DIM-C-pPhOH and DIM-C-pPhCO_2_Me was attenuated after cotreatment with the antioxidant glutathione (GSH). Thus, induction of ROS by NR4A1 antagonist-mediated downregulation of TXNDC5 and IDH1 also results in the induction of sestrin 2 in cells expressing mutant p53, suggesting that NR4A1 antagonists inhibit mTOR by both p53-dependent and-independent induction of sestrin2. These studies demonstrate that NR4A1 is a target for NR4A1 antagonists in RCC cells; this leads to inhibition of several NR4A1-regulated pro-oncogenic pathways (Fig 1A) including mTOR and these results are comparable to those observed in pancreatic, lung and colon cancer cells and tumors [14–17].

## Discussion

NR4A receptors are immediate-early genes that are induced by diverse stressors in multiple tissues/cell lines and these receptors are important for maintaining cellular homeostasis [23, 24]. The function of NR4A1 in cancer cells has also been extensively investigated and in solid tumors, there is evidence that this receptor exhibits unique functions that are dependent on its subcellular location [25–27]. NR4A1 is a nuclear receptor; however, treatment of some cancer cell lines with apoptosis-inducing agents results in the induction and nuclear export of NR4A1 which associates with mitochondrial bcl-2 to form a pro-apoptotic complex [18–20, 25]. Thus, mitochondrial NR4A1 exhibits tumor suppressor-like activity, and both paclitaxel and peptide mimetics of NR4A1 that bind bcl-2 also activate apoptosis in cancer cells [20, 28]. In contrast, results of RNAi studies suggest that nuclear NR4A1 is pro-oncogenic and plays a role in cancer cell proliferation and survival, and studies in this laboratory have identified at least three pathways and associated genes that contribute to the functions of NR4A1 in cancer cells (Fig 1A). Thus, NR4A1 is unique among orphan nuclear receptors, and the observed pro-oncogenic or tumor suppressor-like activity is dependent on the subcellular location of the receptor. Several different structural classes of ligands bind NR4A1 and these include cytosporone B and related analogs [29, 30], C-DIMs [17], ethyl 2-[2,3,4-trimethoxy-6-(1-octanoyl)phenyl]acetate (TMPA) [31], and 1-(3,4,5-trihydroxyphenyl)nonan-1-one [32]. Cytosporone B and related compounds and 1-(3,4,5-trihydroxyphenyl)nonan-1-one induce nuclear export of NR4A1 and TMPA antagonizes nuclear NR4A1-LKB1 interactions but does not affect NR4A1-dependent transactivation. In contrast, our previous studies in pancreatic, lung and colon cancer cells show that C-DIM/NR4A1 antagonists act on nuclear NR4A1 and do not induce nuclear export of the receptor [confirmed in RCC cells (Fig 3)] and these compounds also decrease NR4A1-dependent transactivation [14–17].

Kidney injury can induce NR4A1 expression in non-tumor tissue, and one study reported the NR4A1 was expressed in 786-O cells and NR4A1 mRNA was more highly expressed in tumors from patients with RCC compared to surrounding non-tumor tissue [33–35]. Currently, we are accumulating tumors from kidney cancer patients to investigate the tumor-type specific expression and prognostic significance of NR4A1. We further investigated the pro-oncogenic functions of NR4A1 by RNAi and compared the results with that observed after treatment of ACHN or 786-O cells with two prototypical NR4A1 antagonists, DIM-C-pPhOH and DIM-C-pPhCO_2_Me. Both NR4A1 knockdown and the NR4A1 antagonists inhibited ACHN and 786-O cell proliferation and transactivation, and DIM-C-pPhOH also inhibited tumor growth in athymic nude mice bearing ACHN cells as xenografts (Fig 1) and siNR4A1 and NR4A1 antagonists also induced apoptosis in these cell lines (Fig 2). These effects are comparable to previous studies in pancreatic, lung and colon cancer cell lines [14–17], suggesting the potential therapeutic potential for C-DIMs in treating RCC in patients that overexpress NR4A1.

Fig 1A illustrates three pro-oncogenic pathways and related genes that are regulated by NR4A1 in pancreatic, lung and colon cancer cell lines, and we have also observed these NR4A1-dependent response/pathways in other cancer cell lines (data not shown). NR4A1 in combination with p300 coactivates survivin and other Sp1-regulated genes [14], and results in Fig 4 show that survivin, bcl-2 and the EGFR were regulated by NR4A1 in RCC cells. All three of these Sp-regulated genes play a role in cancer cell growth and survival and some studies show that expression of survivin, EGFR and bcl-2 are negative prognostic factors for patients with RCC [36–41]. Thus, NR4A1 regulation of these genes in RCC contributes to the tumor phenotype, and inhibition of these genes by C-DIM/NR4A1 antagonists in RCC cells and tumors (Fig 4) is an important component of their antineoplastic activity.

A second NR4A1-regulated pathway in cancer cells is associated with regulation of genes such as TXNDC5 and IDH1 that maintain high levels of redox equivalents and decrease intracellular stress [16]. Knockdown of NR4A1 (and NR4A1 antagonists) decreases TXNDC5 and IDH1 and induced ROS and other stress responses in ACHN and 786-O cells (Fig 5), suggesting that NR4A1 also maintains levels of stress that facilitate RCC growth and survival. The functions of TXNDC5 and IDH1 have not been characterized in RCC and it is also possible that NR4A1 regulates other similar genes and this is currently being investigated. Since previous studies show that other ROS-inducing antineoplastic agents inhibit growth and survival of RCC [42, 43], the induction of ROS by C-DIM/NR4A1 antagonists contributed to the anticancer activity of these compounds.

mTOR inhibitors are currently in clinical trials for treating RCC [9–13], and our previous studies showed that in colon and lung cancer cells expressing wild-type p53, NR4A1 inhibits p53 activity and p53-dependent inhibition of the mTOR pathway [15]. However, treatment with C-DIM/NR4A1 antagonists or transfection with siNR4A1 activates p53 which in turn increases expression of sestrin 2 resulting in activation of AMPKα and inhibition of mTOR [15, 17]. These same effects were observed in ACHN cells that express wild-type p53, demonstrating that C-DIM/NR4A1 antagonists represent a novel class of mTOR inhibitors with clinical potential for treating RCC patients expressing NR4A1 and wild-type p53 (Fig 6). Surprisingly, we also observed similar results in 786-O cells that express a mutant p53, and this response was attenuated by cotreatment with glutathione. Sestrin 2 can be induced by oxidative stress [22] and since NR4A1 knockdown and NR4A1 antagonists induce ROS (Fig 5), this represents a second NR4A1-mediated p53-independent pathway that can be targeted by NR4A1 antagonists to inhibit mTOR.

In summary, our results show that NR4A1 is pro-oncogenic in RCC and regulates at least three pathways (Fig 1A) important for cell proliferation and survival, and these can be targeted by C-DIM/NR4A1 antagonists. Our current studies are focused on further investigating the functional responses and molecular pathways regulated by NR4A1 in RCC and identifying RCC patient sub-types that overexpress NR4A1 and are potential candidates for clinical applications of C-DIM/NR4A1 antagonists.

## Supporting Information

S1 Fig Induction of apoptosis. Cells were transfected with siNR4A1 oligonucleotides (A, B) (17) or treated with DIM-C-pPhOH and DIM-C-pPhCO_2_Me (C, D), and whole cell lysates were analyzed by Western blots as described in the Materials and Methods.
(PDF)
