# Supplementary material for: Nuclear Receptor 4A1 (NR4A1) as a Drug Target for Renal Cell Adenocarcinoma
Source: PLoS One. 2015 Jun 2;10(6):e0128308. doi: 10.1371/journal.pone.0128308 (PMC4452731; doi:10.1371/journal.pone.0128308)
Supplement: S1 Fig — Cells were transfected with siNR4A1 oligonucleotides (A, B) (17) or treated with DIM-C-pPhOH and DIM-C-pPhCO2Me (C, D), and whole cell lysates were analyzed by Western blots as described in the Materials and Methods. (PDF) [file pone.0128308.s001.pdf]

Supplemental Figure S1

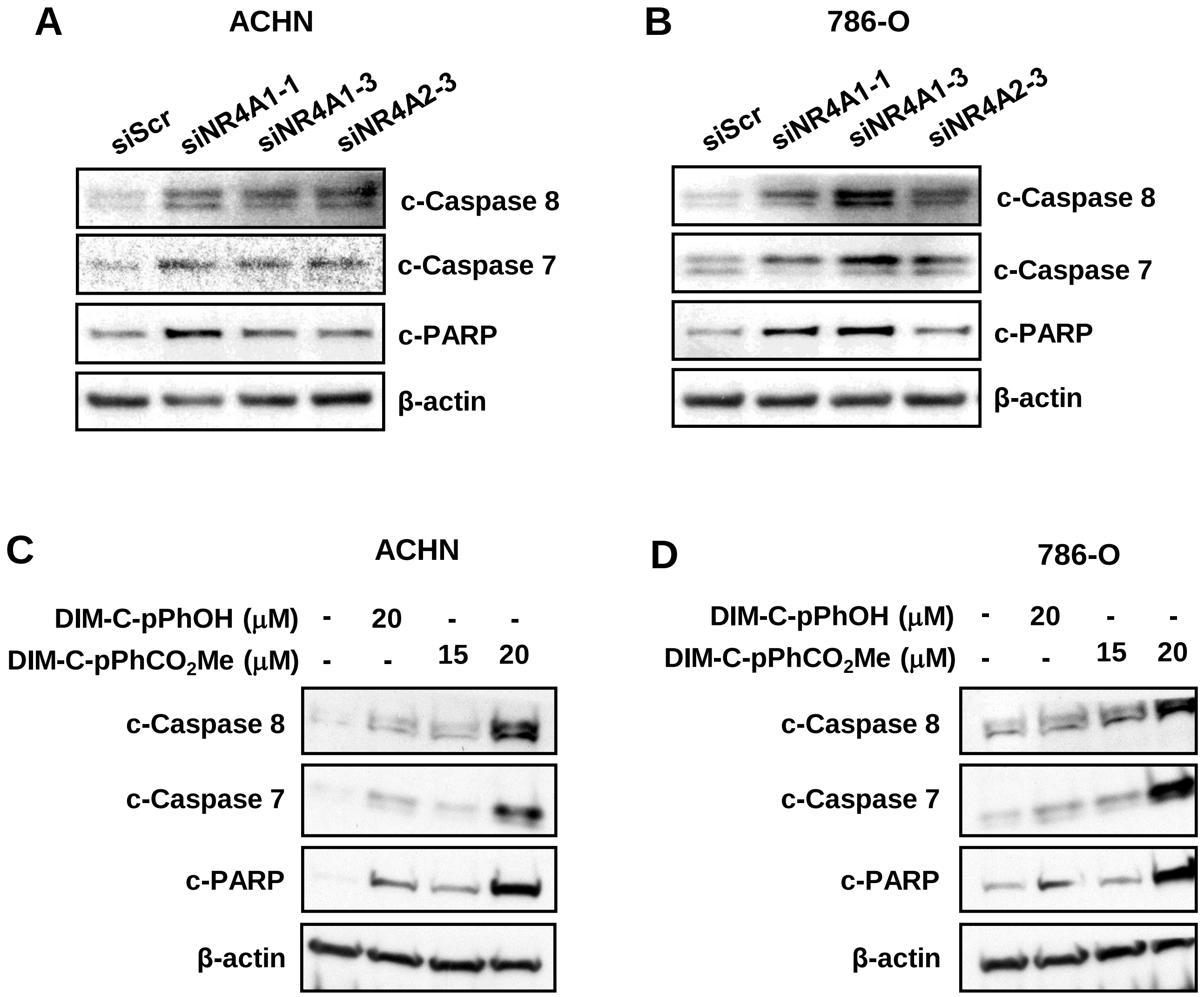

Supplemental Figure S1. Induction of apoptosis. Cells were transfected with siNR4A1 oligonucleotides (A, B) (17) or treated with DIM-C-pPhOH and DIM-C-pPhCO$_2$Me (C, D), and whole cell lysates were analyzed by Western blots as described in the Materials and Methods.
